# Online Indicated Preventive Mental Health Interventions for Youth: A Scoping Review

**DOI:** 10.3389/fpsyt.2021.580843

**Published:** 2021-04-29

**Authors:** Marilon van Doorn, Laurens A. Nijhuis, Mees D. Egeler, Joost G. Daams, Arne Popma, Thérèse van Amelsvoort, Carla McEnery, John F. Gleeson, Ferko G. Öry, Kate A. Avis, Emma Ruigt, Monique W. M. Jaspers, Mario Alvarez-Jimenez, Dorien H. Nieman

**Affiliations:** ^1^Amsterdam University Medical Centers, Amsterdam, Netherlands; ^2^Department of Psychiatry and Neuropsychology, Maastricht University, Maastricht, Netherlands; ^3^Centre for Youth Mental Health, The University of Melbourne, Melbourne, VIC, Australia; ^4^Orygen, Parkville, VIC, Australia; ^5^Healthy Brain and Mind Research Centre and School of Behavioural and Health Sciences, Australian Catholic University, Melbourne, VIC, Australia; ^6^Erasmus University College, Rotterdam, Netherlands; ^7^Minddistrict, Amsterdam, Netherlands

**Keywords:** indicated prevention, mental health, e-health, youth, scoping review, digital, well-being, early detection and intervention

## Abstract

**Objective:** Between the ages of 12 and 25 the onset of mental disorders typically occurs, and the burden of mental health problems is greatest for this group. Indicated preventive interventions to target individuals with subclinical symptoms to prevent the transition to clinical levels of disorders have gained considerable traction. However, the threshold to seek help appears to be high even when help is needed. Online interventions could offer a solution, especially during the COVID-19 pandemic. This scoping review will present an overview of the recent research of indicated online preventive interventions for youth (12–25 years) experiencing the early stages of mental health complaints with the aim of identifying the nature and extent of the research evidence.

**Methods:** The 5-stage framework by Arksey and O'Malley was used. Academic literature published from 2013 onwards in printed or electronic format was included from Scopus, PsychINFO, and Ovid MEDLINE(R) ALL.

**Results:** The search yielded 11,122 results, with the final selection resulting in inclusion of 30 articles for this review. In total, the articles included 4,950 participants. 26.7% of the selected articles focused on youth between 12 and 25 years. Of the articles 60% did not screen for, nor exclude participants with clinical levels of symptoms. Most studies used a common evidence-based therapy for the disorder-category targeted. More than half of the online interventions included some form of human support. Adherence levels ranged between 27.9 and 98%. The results indicate general effectiveness, usability and acceptability of online indicated preventive interventions. The most commonly used approach was CBT (*n* = 12 studies). Studies varied in their size, rigor of study, effectiveness and outcome measures. Online interventions with a combination of clinical and peer moderation (*n* = 3 studies) appear to result in the most stable and highest effect sizes.

**Conclusion:** Online indicated preventive mental health interventions for youth with emerging mental health issues show promise in reducing various mental health complaints, and increasing positive mental health indicators such as well-being and resilience. Additionally, high levels of usability and acceptability were found. However, the included studies show important methodological shortcomings. Also, the research has mainly focused on specific diagnostic categories, meaning there is a lack of transdiagnostic approaches. Finally, clear definitions of- as well as instruments to measure- emerging or subclinical mental health symptoms in youth remain are missing.

## Introduction

Mental health can be defined as “a state of well-being in which every individual realizes his or her own potential, can cope with the normal stresses of life, can work productively and fruitfully, and is able to make a contribution to her or his community” (1). Nonetheless, this state is disrupted in half (2) to almost three quarters (3) of all people living in the western world at some point in their life, and in 1 in 4 in any given year (4). The onset of mental disorders typically occurs in childhood and adolescence (2, 5), with 75% of mental disorders beginning before the age of 25 (5, 6). The waiting lists to receive care accordingly are continually growing (7), and costs associated with mental illness are substantial [e.g., (8)], and growing (9). Moreover, the burden of mental health problems is substantial for these individuals and is indicated by negative effects upon quality of life (10), life expectancy (11) social functioning, ability to work (10), and (self-)stigmatization (10, 12, 13). This burden has been found to be the greatest in young people aged between 15 to 25 years (6).

Increased attention has been paid at interventions aimed at youth with emerging symptoms to treat them as early as possible in the development of a mental disorder, for example, during the peak period of risk for onset, with a focus on both symptomatic as well as functional recovery (14). Prevention and early intervention are recognized as key elements for minimizing the psychosocial and economic impacts of any potentially serious health condition (15, 16). Previous research has shown the effectiveness of face-to-face psychosocial preventive interventions for youth. Improvements in behavioral and social outcomes were observed as well as a decrease in the proportion of participants transitioning from mental health complaints to mental disorders (17, 18). Unfortunately, the gap between needing help and seeking help is substantial. Only one in three young people seek help for their mental health problems (19, 20), and most individuals present to services at a much later stage (21–23). Subsequently these individuals present with more developed and severe problems that are more difficult to treat, and have more functional and social consequences since the mental illness strikes in a critical developmental period where social, vocational and educational milestones were to be achieved (15, 24). In other words, even though help for mental health problems is needed in adolescence or young adulthood, the threshold to seek it appears high. Perceived barriers for help-seeking in young people include negative attitudes toward seeking help (e.g., internalized stigma or shame), practical concerns (e.g., costs and transportation), believing they have to manage the problem on their own, downplaying their problems, doubts concerning the effectiveness of treatment, the unavailability of help (19, 25) and perceived public mental-health stigma (26).

Online interventions might offer a solution to the perceived barriers. Advantages of online interventions are the possibility to receive help anonymously, and increased convenience because individuals can choose when and where they access help (27–29). Moreover, online interventions have the potential to reach people who are unwilling or unable to receive face-to-face help, for example, those who live in remote areas or those with decreased mobility (30). Online interventions may be especially appealing to young people as most youth are familiar and competent with using digital technology. This is illustrated by data that indicate that 96% of European youth (aged 16–24) use the internet regularly (31). Furthermore, research shows that young people report using the internet to find information pertaining to mental health (32), they have positive perceptions about using the internet for mental health related-issues (33), and clinicians hold positive attitudes toward using technology for treatment too (34). Moreover, online interventions hold the potential to decrease costs for the individual and the healthcare system. Lastly, online interventions offer mental health care from home during the current COVID-19 pandemic (35, 36) which may be especially important for individuals with emerging complaints who are prone to developing more severe mental health issues (37).

Online indicated preventive interventions for individuals with an indicated need for care, that is, youth with emerging complaints, offer a promising approach to address this unmet need. From a resource perspective, it may be more feasible to target individuals with subclinical symptoms than non-symptomatic individuals who may not have a need for an intervention (38). The clinical staging model (39, 40) illustrates the differentiation between subclinical symptom clusters (stage 1a or 1b) and the onset of more discrete syndromes or clinical entities (stage 2, 3, and 4). Previous meta-analyses investigating face-to-face preventive interventions in youth have also shown that indicated preventive interventions have larger intervention effects than universal preventive interventions (38, 41).

The effectiveness of online indicated mental health preventive interventions for young people has been addressed in four systematic reviews and two meta-analyses between 2014 and 2016. In these reviews “youth” is defined as between the ages of 12 and 25, in concordance with most international definitions of youth as well as governmental and youth mental health institutions (42, 43). Interestingly, the majority of participants included in these reviews are youth with subclinical symptoms (stage 1a or 1b), however some participants might be in a later stage since a clinical diagnosis was not an exclusion criterion in most included studies. Also, the transition to clinical disorders, which is the established primary outcome of indicated preventive treatment trials, was generally not measured. Lastly, the reviews varied quite substantially in their scope. Rice et al. (44) conducted a systematic review including studies focusing on online and social networking as indicated preventive interventions for the treatment of depressive symptomatology in youth (12–25 years). The overall finding was that online interventions appear to be promising in reducing depression symptomatology in young people. The systematic review of Ali et al. (45) included six studies targeting online peer-to-peer support for young people (12–25 years) with emerging mental health problems. Two out of six studies found support for the effectiveness of online peer-to-peer support although an overall lack of quality of the studies was found, and the type of moderation used in the studies was poorly reported. In 2015, Pennant et al. (46) included 27 studies in their systematic review and meta-analysis researching both indicated and universal preventive computerized therapies for anxiety and depression in children and young people (12–25 years). It was found that indicated and general preventive intervention had positive effects for reducing symptoms of anxiety and depression. However, follow-up data about long-term effects was scarce, and the authors stated that the magnitude of the effects needed to be interpreted cautiously due to the heterogeneity associated with a number of outcomes and predominantly low quality of the evidence. In all three systematic reviews it was not specified whether the indicated prevention had an effect on the rate of transition to clinical disorders, since outcome measures included solely measures of symptom severity.

O'Dea et al. (47) reviewed the evidence for online interventions for universal and indicated prevention targeting depression and anxiety symptoms and disorders in youth (12–18 years). They included six studies, and found positive effects on symptoms in all but one trial. They concluded that there are a number of gaps in the literature, for example a lack of cost-effectiveness data, and heterogeneity in sample sizes, randomization procedures, and outcome measures, making it difficult to compare trial results. There was only one study that measured the effect of indicated prevention on the development of clinical levels of depression; it was found that Cognitive Behavioral Therapy (CBT) lowered this risk. Ebert et al. (48) conducted a meta-analysis including internet and computer-based cognitive behavioral therapy for anxiety and depression in children and youth (< 25 years). They included 13 Randomized Control Trials (RCTs) and found an overall effect size of *g* = 0.72, reflecting a decrease in symptom severity. Again, the authors reported high heterogeneity and long-term effects of the studies. In the most recent meta-analysis of Conley et al. (49) the impact of universal and indicated preventive technology-delivered interventions for higher education students (age not specified) was investigated. They included 22 universal and 26 indicated prevention studies, and found larger positive treatment effects for indicated preventive interventions than universal preventive interventions. The authors reported important limitations on the experimental rigor and recommended that future research should for example provide more details on participant characteristics, and intervention content; and collect follow-up data.

While Rice et al. (44), Ali et al. (45), Pennant et al. (46), O'Dea etal. (47), Ebert et al. (48), and Conley et al. (49) included online indicated preventive interventions in their reviews, universal preventive interventions were included as well. To our knowledge no more recent reviews have been published. Moreover, there have been no reviews specifically of studies focusing on the effect of indicated preventive interventions provided online for youth. This scoping review will present an overview of the recent research of indicated online preventive interventions for youth experiencing the early stages of mental health complaints with the aim of identifying the nature and extent of the research evidence.

## Methods

### Framework

We utilized the 5-stage framework by Arksey and O'Malley (50) developed for reporting a scoping review. This framework entails the following stages: (1) identifying the research question, (2) identifying relevant studies, (3) selection of studies, (4) charting the data, and (5) summarizing and reporting the results.

### Research Question

The focus of this review was to present an overview of indicated online preventive interventions for emerging mental health symptoms in youth and aimed to identify the nature and extent of the research evidence. This led to the following guiding question: *What is known in the literature about the use of indicated online preventive interventions for youth with emerging mental health problems?*

### Search Strategy

A search was conducted together with the university librarian with experience in conducting reviews (JD). Appendix 1 displays the used search terms. Academic literature published in printed or electronic format was included from the following sources: Scopus, PsychINFO, and Ovid MEDLINE(R) ALL. Articles written in the English and Dutch language were retrieved. In order to ascertain recent findings, articles from 2013 onwards were included in this review. Study designs were limited to randomized controlled trials, quasi-experimental study designs and experimental studies without a comparison group. See Supplementary Material for the search criteria.

### Eligibility Criteria

The eligibility criteria were determined to find all articles relevant to the research question. A highlighted summary of the main inclusion/exclusion criteria covered here is provided in Table 1.

**Table 1:** Eligibility criteria.

	**Inclusion**	**Exclusion**
Population	Youth (majority included 12–25 years old) Signs or symptoms of a mental health disorder	Children (<12 years old) and adults (>25 years old). Known mental health disorder
Intervention	Online prevention interventions (online-, internet-, web-, or mobile-based) Online *indicated* preventions	Interventions primarily face-to-face, Universal prevention, Selective preventive interventions
Comparison	Online prevention program, website, app, game, social media or smartwatch intervention compared to intervention, waiting list, or face-to-face intervention	–
Outcomes	Negative mental health indicators Positive mental health indicators Well-being indicators	Outcomes that are not indicative of mental health and well-being
Timing and setting	From 2013 onwards	–
Language	Articles written in English or Dutch	Other
Study design	RCTs, Quasi-experimental study designs, Experimental studies without comparison group	Descriptive studies, protocols

### Population

Studies eligible for inclusion were those containing a sample of youth, defined in concordance to most international, governmental and youth institutional definitions (42, 43) as participants aged 12–25, who have signs or symptoms of a mental disorder that are either self-reported or assessed via a screening process. Studies were included if they included participants below age 12 or above age 25 as long as the majority of the participants was between ages 12–25. This was assessed by the mean age of the participants, the standard deviation of age, and the proportion of participants within this age range (studies were included only when more than half of the participants fell within the range). Articles were excluded when the age range of the included participants was outside 12–25 and the mean age was not reported.

Studies were also excluded if they had selected participants with a known mental disorder (either self-reported or diagnosed by a clinician). However, since most indicated preventive interventions did not screen for the presence of a mental disorder, studies were not excluded if they did not screen for mental disorders. The risk of reducing specificity by including these articles was deemed essential in order to have a broader scope to best summarize the relevant research.

### Intervention

Interventions needed to be delivered primarily in an online (digital) setting (defined as: online-, Internet, Web-, or mobile-based) and focused on indicated prevention of mental disorders in youth aged 12–25 with signs or symptoms of a mental disorder. Only online indicated preventive interventions were included [defined as: preventive online interventions which target individuals who are showing early symptoms and signs of a disorder to prevent progression from clinical stages 1a and 1b to stages 2–4; (40)].

Interventions that are primarily face-to-face with some additional online content were also excluded. Lastly, process evaluation studies were excluded (although important implementation findings may be highlighted in the identified studies).

### Comparison and Study Type

Studies comparing online prevention programs, websites, apps, games, social media or smartwatch interventions compared to no intervention, waiting list, or face-to-face interventions were included. No exclusion criteria were applied to the type of comparator used. RCTs, quasi-experimental study designs and experimental studies without a comparison group were eligible for inclusion in this review. No minimum follow-up time period was specified. Observational studies or protocols were excluded. There were no restrictions by timing or type of setting. Only articles written in English or Dutch were included.

### Outcomes

The following a priori determined outcome measures were included: negative mental health indicators, for example, depression, anxiety, psychological distress and suicidal behavior, and transition to symptom levels above clinical diagnostic threshold; positive mental health indicators, for example, self-efficacy, coping skills, resilience, emotional well-being, self-esteem; and well-being indicators, for example, social participation, quality of life, social functioning, empowerment, communication, social support. Outcomes that were not indicative of mental health and well-being were excluded.

### Charting the Data

Data were extracted by two out of five reviewers independently and in duplicate with the use of standardized data extraction forms. Any disagreements were resolved through discussion and by a third reviewer. To ensure accuracy, the extracted data was reviewed by experts in the field based on relevance. Data that was extracted included: intervention characteristics, methodology, program outcomes and information about program completion, engagement rate, and inclusion of human support.

The following data items were extracted: (1) intervention name, author, country where research was conducted, year of publication, (2) intervention characteristics (type of intervention, duration, target group), (3) method (study design, sample, selection biases, confounders, blinding, data collection, analysis, intervention integrity), (4) program outcomes, and (5) adherence (non-completion/dropout rates). In case of deficient or missing outcome data, authors were contacted and data were requested.

### Summarizing and Reporting Results

To determine the extent and nature of the studies, a numerical analysis was conducted, using tables and chart mappings. Using conventional content analysis the descriptive data was analyzed. The user-centered design framework was followed, which states that two reviewers have to examine the data and identify codes relative to the findings. The codes were grouped according to themes to summarize the literature and answer the research question.

## Results

**Figure 1:**
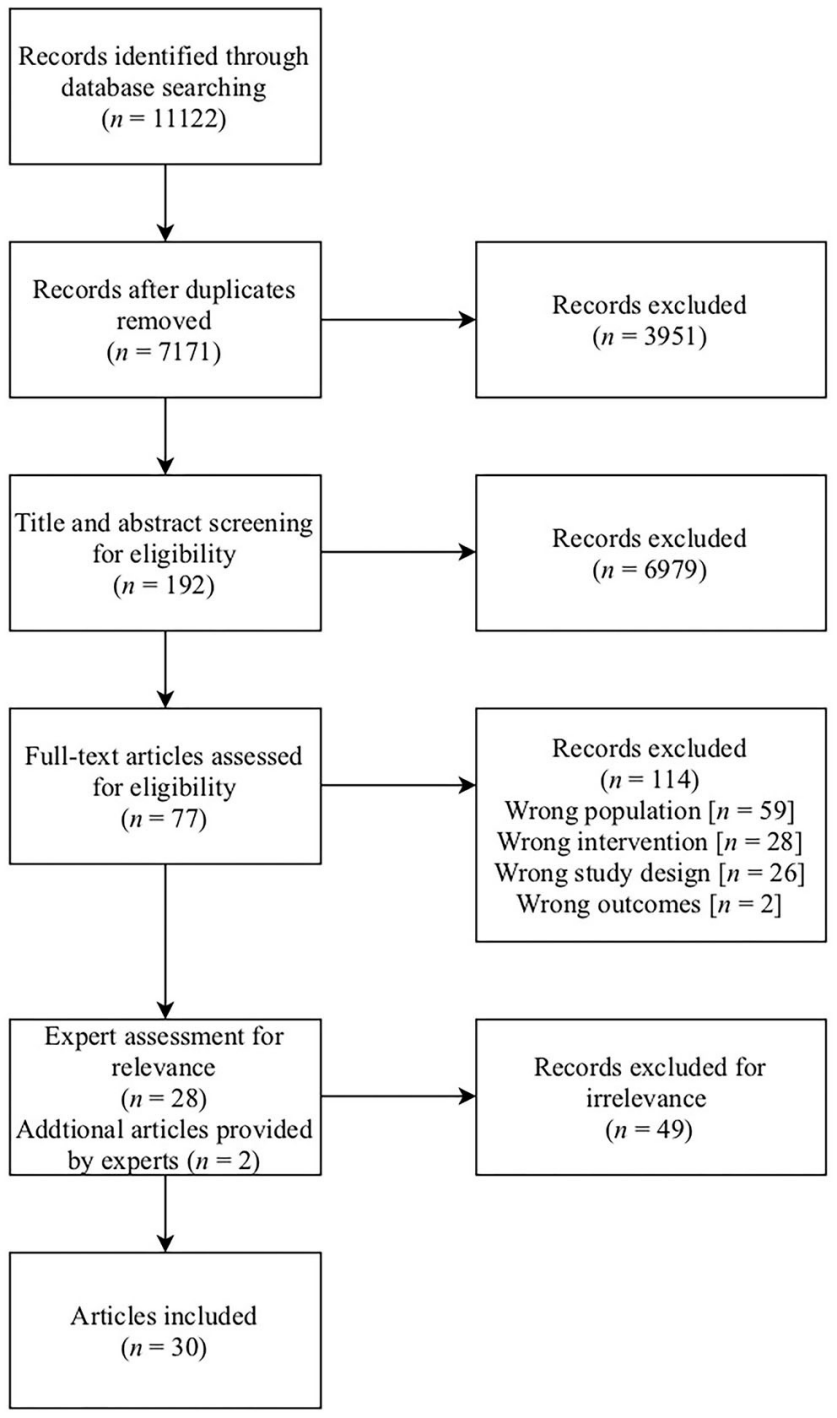
PRISMA diagram.

The search conducted by the university librarian (JD) yielded 11,122 results. After the screening process, the remaining 77 articles were assessed for relevance using the eligibility criteria in Table 1 by six experts (DN, MAJ, JG, TvA, AP, and CM), and an additional two articles were provided by experts, resulting in the final inclusion of 30 articles for this scoping review. For the study selection procedure, see the PRISMA diagram in Figure 1.

For all study characteristics, see Table 2.

**Table 2:** Study characteristics.

**Author**	***N***	**Mean age (SD)**	**Gender**	**Type of complaints**	Disorder excluded?^*^	**Adherence**	**Location**
Alvarez-Jimenez et al. (51)	14	20.3 (3.4)	78% female	At risk for psychosis	CAARMS	57% participants completing at least 6 therapy modules and 43% completing 9 or more therapy modules	Australia
Alvarez-Jimenez, et al. (52)	157	19.1 (2.3)	77% female	Self-reported mental health concerns	No	Unclear	Australia
Anttila et al. (53)	46	16 (?)	74% female	Depressive or anxiety symptoms	No	100% adherence (*n* = 5 withdrew before start, *n* = 24 non-response)	Finland
Aubel et al. (54)	55	21 (2.43)	73% female	Depressive or psychotic symptoms	Currently under treatment or need for more care assessed by psychiatrist	92.59%	Netherlands
Berg et al. (55)	71	17.2 (1)	94% female	Depressive symptoms	No	70.0% fully completed, on average 81% of 8 modules	Sweden
Cook et al. (56)	235	20.41 (?)	83% female	Depressive symptoms	Instrument not specified	Unclear	UK
Deady et al. (57)	60	21.74 (2.22)	60% female	Depressive symptoms and alcohol use	No	63.3%	Australia
Dickter et al. (58)	83	17.5 (2.04)	56.2% female	Depressive symptoms	PHQ-A interview	26.5% no modules, 24.1% 1–5 modules, 20.5% 6–13 modules, 28.9% entire program	USA
Farrer et al. (59)	200	22 (4.1)	77.5% female	Transdiagnostic	No	75.8% used program at least once	Australia
Fitzpatrick et al. (60)	70	22.2 (2.33)	67% female	Depressive/anxiety symptoms	No	52% (used provided e-book at least once)	USA
Harrer et al. (61)	150	24.1 (4.1)	74.7% female	Elevated stress	No	71.2%	Germany
Hickie et al. (62)	449	?	63% female	Distress symptoms	No	Weekly use 18%, 1–2 times a month or less 82%.	Australia
Hides et al. (63)	169	19.9 (2.5)	79.3% female	Distress symptoms	No	54.44%	Australia
Hill et al. (64)	80	16.67 (1.7)	68.8% female	Suicide prevention and burdensomeness	No	43.90%	USA
Hullu et al. (65)	240	13.6 (?)	72.5% female	Social anxiety symptoms	No	41.86%	Netherlands
Lattie et al. (66)	39	16.23 (.99)	74.4% female	Depressive symptoms	M-health history assessed by psychiatrist	Unclear	USA
Levin et al. (67)	79	20.51 (2.73)	66% female	Transdiagnostic: in distress	No	55% completed all, 75% completed half, 17.5% didn't participate.	USA
Levin et al. (68)	234	21.61 (5.48)	76.9% female	Depressive, anxiety and distress symptoms	Self-report no former diagnoses	ACT: 1st lesson completed 85%, 2nd lesson completed 55% - Online Education 1st lesson completed 100%, 2nd lesson completed 86%	USA
Mccall et al. (69)	65	21.86 (5.51)	72% female	Social anxiety symptoms	Self-report no former diagnoses	98.0%	Canada
Mcdermott et al. (70)	350	18.75 (1.63)	73.2% female	Depressive symptoms	No	77.2% completed	Canada
Motter et al. (71)	46	21 (3.7)	71.7% female	Depressive symptoms	No	76.09%	USA
Poppelaars et al., (72)	208	13.35 (.71)	100% female	Depressive symptoms	No	95.39%	Netherlands
Radomski et al. (73)	536	16.6 (1.7)	71.3% female	Anxiety symptoms	No	27.90%	Canada
Sanci et al. (74)	413	20.7 (2.3)	83.3% female	Symptoms of distress and negative affect	No	71% responded to 2-week, 1-month, 3-month follow-up.	Australia
Simmons et al. (75)	66	18.5 (3.42)	82% female	Depressive symptoms	Instrument not specified	76.0%	Australia
Staples et al. (76)	424	21.5 (2)	82.8% female	Depressive or anxiety symptoms	PHQ-9	100% 1 lesson, 88% 2 lessons, 79% 3 lessons, 64% completed all (4) lessons	Australia
Takahashi et al. (77)	22	20 (.62)	27.3% female	Depressive symptoms	Self-report no former diagnoses	50.0%	Japan
Topper et al. (78)	251	17.45 (?)	83.7% female	Depressive or generalized anxiety disorder symptoms	Self-report no former diagnoses + PHQ-9	86.83%	Netherlands
Traber-Walker et al. (79)	30	16.1 (?)	61% female	At risk for psychosis	Self-report no former diagnoses	-	Switzerland
De Voogd et al. (80)	108	14.45 (1.53)	66.7% female	Depression and anxiety symptoms	No	43.8%	Netherlands

* *This section specifies whether there was screened for known mental disorders, and if people with a mental disorder were excluded*.

*SD, standard deviation; PHQ-9, patients health questionnaire-9; PHQ-A, patients health questionnaire-9 modified for adolescents; m-health, mental health; CAARMS, comprehensive assessment of at risk mental states; CBT, cognitive behavioral therapy; IPT, interpersonal therapy; ACT, acceptance and commitment therapy*.

### Timeline

Six studies were published in 2016. The number of studies regarding preventative online measures for youth saw a slight decline in the years 2017 (*n* = 5) and 2018 (*n* = 4), followed by a spike in publications occurring in 2019 (*n* = 13). At the present moment, there have been an additional two studies published in 2020.

### Geographic Location

The included studies were predominantly conducted in Australia (*n* = 9) and the United States of America (*n* = 7), accounting for more than half (53%) of the contributions in this scoping. The remaining studies were conducted in the Netherlands (*n* = 5), Canada (*n* = 1), Finland (*n* = 1), Germany (*n* = 1), Japan (*n* = 1), Sweden (*n* = 1), Switzerland (*n* = 1), and the United Kingdom (*n* = 1). See Table 2.

### Study Design

The included studies consisted primarily of RCTs (*n* = 23). Of these 23 RCTs, two were stratified and one was clustered. The remaining were experimental designs without comparison groups (*n* = 6). Another study that was included based on the preliminary inclusion criteria was identified as a mixed design (*n* = 1). Almost half (46%) of all included studies made use of a follow-up procedure, either within 3 months (*n* = 4), between 3 to 6 months (*n* = 6), or after 7 months and beyond (*n* = 4). See Table 3.

**Table 3:** Study outcome measures and results.

**Author**	**Study design**	**Intervention type**	**Duration of intervention**	**Outcome measures**	**Human support**	**Findings**
Alvarez-Jimenez et al. (51)	Experimental study without comparison with 2-month follow-up	Mindfulness and strength based intervention	2-months	Psychotic Symptoms, Depression, Stress, Social and Global Functioning, Mindfulness, Personal Strengths, Social Provision, Life Satisfaction, Self-Efficacy, Self-Esteem, Loneliness	Peer and clinically moderated	Large improvement in social functioning (*d* = 1.83), subjective well-being (*d* = 0.75), strengths usage (*d* = 0.70) and mindfulness skills (*d* = 0.66) at follow-up.
Alvarez-Jimenez, et al. (52)	Experimental study	CBT, mindfulness, self-compassion and positive psychology	9 weeks	Nonspecific Psychological Distress, Mental Well-being, Stress, Depression, Loneliness, Psychological Needs, Friendship Strength, Use of Strength, Mindfulness, Platform Usability	Peer and clinically moderated (clinician guidance, chat counseling, and peer moderators)	Improvements in psychological distress (*d* = −0.38), perceived stress (*d* = −0.37), psychological well-being (*d* = 0.38), loneliness (*d* = −0.33), social support (*d* = 0.25), and autonomy (*d* = 0.50).
Anttila et al. (53)	Mixed Methods descriptive study	Self-determined by participants	6 weeks	Quality of Online Services	Clinically moderated (feedback on exercises by research nurse)	89% rated web-based support system reliable and safe. 93% rated the content on web-based support systems relevant
van Aubel et al. (54)	RCT with 6 and 12-month follow-up	ACT	6 weeks	Depression, Psychotic symptoms, Anxiety, General psychopathological symptoms, Psychological Flexibility, Negative and Positive Affect	Clinically moderated (weekly group sessions with trained therapist)	Decrease in depressive symptoms (*p* = 0.027) compared to control. Increased mean negative affect (*p* = 0.011), relative to active controls.
Berg et al. 2019	RCT	CBT	8 weeks	Depression, Psychological Knowledge	Clinically moderated (weekly chat contact and feedback on exercises)	Improvements in psychological knowledge in ICBT compared to attention control (between-group *d* = 1.25). Non-significant correlation between change scores in knowledge and BDI-II change scores
Cook et al. 2019	RCT with 3- and 15-month follow-up	CBT	6–12 weeks	Depression, Anxiety, Worrying, Rumination	Clinically moderated (feedback by clinicians)	Reduced risk of depression by 34% using guided i-RFCBT relative to usual care (hazard ratio = 0.66). Significant improvements in rumination, worry, and depressive symptoms.
Deady et al. (57)	RCT	CBT and motivational interviewing	4 weeks	Depression, Alcohol use	None	Reduction in depressive symptoms (*d* = 0.71; 6-month follow-up: *d* = 0.39), reductions in alcohol use quantity (*d* = 0.99; 6-month follow-up: *d* = −0.09) and frequency *d* = 0.76; 6-month follow-up: *d* = 0.24) compared to control.
Dickter et al. (58)	Experimental study, without comparison	CBT and IPT	Not specified	Suicidal ideation, Hopelessness, Low self-esteem, Social isolation	None	Decrease in suicidal ideation (*d* = 0.60).
Farrer et al. (59)	RCT	Psychoeducation, CBT and mindfulness	6 weeks; young people choose amount of modules	Depression, Anxiety, Non-specific Psychological Distress, Social Anxiety, Quality of Life, Self-efficacy, Academic Self-Efficacy	None	Reductions social anxiety (*d* = −0.03; 3-month follow-up *d* =-0.17). Improvements in academic self-efficacy (*d* = 0.10; 3-month follow-up *d* = 0.60).
Fitzpatrick et al. (60)	RCT	CBT	2 weeks	Depression, Anxiety, Positive and Negative Affect	Robot	Decreased depressive symptoms in the Woebot condition (*d* = 0.44) over control. Reduced anxiety symptoms in both groups (*d* = 0.37)
Harrer et al. (61)	RCT with 3-month follow-up	CBT and 3rd wave techniques	5–7 weeks	Perceived stress, Depression, Anxiety, Well-being, Emotional exhaustion, Dysfunctional perfectionism, Resilience, Self-compassion, Self-esteem, Academic work impairment, Academic productivity, Academic self-efficacy, Academic worrying	Clinically moderated (guidance by psychology student; check adherence, give feedback on exercises)	Improvements in stress (*d* = 0.69), anxiety (*d* = 0.76), depression (*d* = 0.63), college-related productivity (*d* = 0.33), academic work impairment (*d* = 0.34) compared to control. Effects remained at follow-up
Hickie et al. (62)	Experimental study without comparison with 15, 30, 60, 90 day follow-up	Decision aid for treatment	90 min	Non-specific Psychological Distress, Suicidality, Personal concerns, Positive Mental Health, Happiness, Program Usability	Clinically moderated (a health professional present)	Significant reduction in psychological distress, body image issues, depression, and coping with stress. Improvement in health and mental health rating.
Hides et al. (63)	Stratified RCT with 1, 2, 3, 6-month follow-up	Music therapy	1-month	Emotion Regulation, Non-specific Psychological Distress, Positive Mental Health	None	Significant improvements in 5 of the 6 emotion regulation skills, mental distress, and well-being at 2, 3, and 6-months. No significant differences between groups
Hill et al. 2016	RCT with 6 week follow-up	CBT	2 weeks	Interpersonal Needs, Perceived Burdensomeness, Thwarted Belongingness, Depression, Suicide Ideation, Satisfaction with Services	None	Lower perceived burdensomeness scores (partial η^2^ =0.10; follow-up: partial η^2^ = 0.21), lower depressive symptoms (follow-up: partial η^2^ = 0.12), and lower thwarted belongingness (follow-up: partial η^2^ = 0.16) compared to control.
de Hullu et al. (65)	Clustered RCT	Cognitive bias modification internet-based vs. CBT f2f	10 weeks	Social phobia, Test anxiety, Self-esteem, Prosocial behavior, Fear of Negative Evaluation, Self-esteem, Implicit Cognition	None	Decrease in social and test anxiety (2-year follow-up: *d* = 0.86, and 0.82 respectively). Positive changes in self-esteem (*d* = −0.67), prosocial behaviors (*d* = −0.57), and fear of negative evaluation (*d* = 0.49).
Lattie et al. (66)	RCT	CBT	8 weeks	Depression, Positive Affect, Perceived Stress, Alcohol and Drug use, System Usage and Usability	Peer and clinically moderated (for guidance and technical support)	Decreased depressive symptoms ($\eta_{p^{2}}$ = 0.061) and perceived stress ($\eta_{p^{2}}$ = 0.159); significant increase of positive effect from baseline to midpoint in both groups ($\eta_{p^{2}}$ = 0.321)
Levin et al. (67)	RCT	ACT	4 weeks	Depression, General Anxiety, Social Anxiety, Academic Concern, Eating Disorder Symptoms, Hostility, Alcohol use, Distress, Psychological Inflexibility, Positive mental Health, Personal values, Mindfulness, Cognitive fusion, Program Usability	None	Decrease in total distress (*d* = 0.66), social anxiety (*d* = 0.78), academic concern (*d* = 0.62), MHC total score, (*d* = 0.58), and MHC social well-being (*d* = 0.69). Significant time by condition interactions were found for PHLMS acceptance (*d* =0.053), and VQ obstacles (*d* = 0.65)
Levin et al. (68)	RCT with 3 week follow-up	ACT	3 weeks	Depression, Anxiety, and Stress Symptoms, Psychological Inflexibility, Positive Mental Health, Personal values, Relationship and Education, Mindfulness, Knowledge of ACT core concepts, Program Usability	None	No differences between conditions at post or follow-up.
Mccall et al. (69)	RCT with 4-month follow-up	CBT	4-months	Social Anxiety, Fear of Negative Evaluation, Quality of life	None	Reduction in social anxiety in treatment condition (SIAS: *d* =0.72; FNE: *d* = 0.82) then control condition (SIAS: *d* =0.56; FNE: *d* = 0.97)
Mcdermott et al. (70)	RCT	CBT vs. attention bias modification	6 weeks	Neuroticism, Non-specific Psychological Distress, Depressive Symptoms Anxiety and Stress	None	Greater improvement in depressive symptoms in CBT condition vs. attentional bias modification (*d* = 0.37 and *d* = 0.48; follow-up: *d* = 0.57 and *d* = 0.65).
Motter et al. (71)	RCT	Cognitive training	8 weeks	Depression, Social dysfunction, Letter Fluency, Cognitive Flexibility	None	Greater improvement in coding (*d* = 0.45), executive functioning and processing speed in EF/PS group compared to verbal group. Improvements in self and clinician-rated depressive severity, everyday functioning, and cognition in both groups.
Poppelaars et al. 2016	RCT with follow up at 3-, 6-, 12-month interval	CBT	8 weeks	Depression	Clinically moderated (in one condition combined face-to-face therapy with e-health)	Decrease depressive symptoms in all conditions (*p* < 0.001; 1-year follow-up: partial η^2^ = 0.14), no difference between conditions.
Radomski et al. (73)	RCT with 6 week follow-up	CBT	6 weeks	Anxiety, Experience of E-Health Interventions	Clinically moderated (one coaching session by clinician)	Total user experience was significantly more positive for the interactive online platform than for respondents using a webpage.
Sanci et al. (74)	RCT with 1 and 3-month follow-up	Decision aid	Not specified	Non-specific Psychological Distress, Positive and Negative Affect, Help-Seeking Behavior	None	Decrease in negative effect compared to control (*p* = 0.02; 1-month follow-up: *p* = 0.001). Increase in help seeking behavior compared to control (3-month follow-up: *p* = 0.04
Simmons et al. (75)	Experimental study without comparison	Decision aid for treatment and life style advice, and psychoeducation	50 min	Depression, Decisional conflict, satisfaction with decision	Clinically moderated (clinician present during session)	Clients were more likely to make a guideline congruent decision for treatment (93 vs. 70%; *P* = 0.004), had reduced decisional conflict and reduced depressive symptoms (follow-up: 7 points lower on PHQ).
Staples et al. (76)	RCT using data from an already completed study	CBT	8 weeks	Depression, Anxiety, Non-specific Psychological Distress, Treatment Satisfaction	Clinically moderated (one condition with support clinician)	Symptom reductions on all measures at post-treatment and 3-month follow-up both conditions. Within-group effect sizes were large (*d* >1.0) and high levels of treatment satisfaction.
Takahashi et al. (77)	Experimental study without comparison	Motion picture-reproducing app	5 weeks	Depression, General Mental Health, Social Anxiety, Self-Efficacy, Salivary Interleukin-6 levels, Program Usability	None	Decrease depressive symptoms (*d* = 0.94).
Topper et al. 2017	RCT with 3-month and 12-month follow-up	CBT	6 weeks	Worrying, Rumination, Perseverative Thinking, Depression, General Distress, Mood and Anxiety, Eating Disorder Symptoms, Alcohol Use	Clinically moderated (weekly group session and feedback on online exercises by a therapist)	Reduced RNT (*d* = 0.53 to 0.89; 12-month follow-up: effects maintained), and symptoms of anxiety and depression (*d* = 0.36 to 0.72; 12-month follow-up: effects maintained) in both interventions. Significantly lower 12-month prevalence rate of depression and generalized anxiety disorder in both intervention groups compared to the waitlist
Traber-Walker et al. (79)	RCT	Adjunct to therapy; e.g. information, registrations	16 weeks	Global and Social Functioning, Quality of Life, Self-Efficacy, Treatment Satisfaction	Clinically moderated (weekly individual sessions)	Ongoing
de Voogd et al. (80)	Stratified RCT with 6-month follow-up	Attentional bias modification	4 weeks	Anxiety, Depression, Perseverative Thinking, Mental Recognition Task, Strengths and Difficulties, Self-Esteem, Emotional-Visual Cognition, Cognitive Recognition	Clinically moderated (sending of reminders and technical support)	Reductions in symptoms of anxiety and depression; and an increase in emotional resilience. Attentional bias modification reduced attentional bias compared to both control groups.

*N, number of participants; RCT, randomized clinical trial; ACT, acceptance and commitment therapy; CBT, cognitive behavioral therapy; d, Cohen's d; ηp2, partial eta- squared; p, p-value; PHQ, patient health questionnaire; EF/PS, executive functioning and processing speed; ICBT, internet-based cognitive behavioral therapy; BDI-II, becks depression inventory II; MHC, mental health continuum; PHLMS, the philadelphia mindfulness scale; VQ, valuing questionnaire; SIAS, Social Interaction Anxiety Scale; FNE, the fear of negative evaluation scale; RNT, repetitive negative thinking; i-RFCBT, internet-based rumination-focused cognitive behavioral therapy; f2f, face-to-face*.

### Sample Size and Study Population

See Table 2 for sample characteristics. In total, the 30 articles included 4,950 participants in their studies, (165 participants per study on average), ranging from 14 to 536 participants. In total, 26,7% of the selected articles focused on young people between the ages of 12 and 25, 30% focused solely on youth from the ages 11 to 19, and 43,3% focused on adolescents of 17 years and older. The weighted mean age of participants over all articles was 18,9 years. In all articles but 1 (77), the majority of participants were female. 33,3% of the articles focused on depression symptoms or disorders, 10% on anxiety related symptoms or disorders, and 20% on either depression or anxiety symptoms or disorders. 6,7% of the articles focused on symptoms of psychosis, 3,3% on suicide, and 3,3% on depression or psychosis symptoms. 23,3% of the articles focused on elevated stress and had a transdiagnostic approach.

Of the included articles 40% focused solely on indicated prevention and excluded participants who met criteria for a mental disorder. The other 60% did not screen for, or exclude participants with presence of a mental disorder. Therefore, these studies were not strictly indicated prevention studies despite using the terms “prevention” or “indicated” in the publications. The measures used to establish whether a participant had emerging complaints vs. a known mental disorder varied substantially over the studies, ranging from self-report (e.g. “Have you ever been diagnosed with a mental disorder?”) to a structured DSM-5 interview with clear cut-offs for clinical levels of mental disorders. A specific and validated clinician-rated instrument was used only for identifying the subclinical complaints of psychosis (UHR-state) (51). Lastly, the studies used different at-risk definitions, which indicates that a clear consensus on definitions is also missing.

### Intervention Type and Duration

Most of the studies used a common evidence-based therapy for the disorder-category targeted. The most commonly used approach was CBT (*n* = 17), of which several studies (*n* = 5) combined this approach with another, for example Interpersonal Therapy (58), Motivational Interviewing (MI) (57), third wave techniques (61), mindfulness (59), and strength-based interventions such as mindfulness, self-compassion and positive psychology (52). One study researched Cognitive Training (CT) (71), and another mindfulness and strength training (51). Three studies investigated Acceptance and Commitment Therapy (ACT) (54, 67, 68). One study used cognitive bias modification (65), and two attentional bias modification (70, 80). Less common approaches were used by Takahashi et al. (77) using a motion picture producing app; Anttilaet al. (53) who used self-determination as a framework and allowed participants chose relevant subjects to discuss; and Hides et al. (63) using Music Therapy. In the study by Traber-Walker et al. (79), an app was used as an adjunct to face-to-face therapy; for example, containing information and registration forms. Lastly, three studies offered a decision aid to help find the right treatment, and find information (e.g., lifestyle advice, psychoeducation); and did not provide further treatment on their platforms (62, 74, 75). More than half of the online interventions included some form of human support, ranging from sending reminders, to group or individual sessions with clinicians. In most studies the treatments were based on specific theoretical bases for the disorder-category being targeted, for example, CBT for depressive symptoms, with standard modules and options to tailor the treatment to the individual's needs. See Table 3.

The range of duration of the online intervention was 50 min to 16 weeks. See Table 3. The three decision aid programs had the shortest duration, namely only one session (50 or 90 min; and not specified). Not taking these three studies into account, the online treatment programs varied in duration from 2 weeks to 16 weeks.

### Adherence

The included studies showed a varied range of adherence to the programs, see Table 2. The adherence percentages were either adopted directly from the reported number provided by the authors of the included papers, or calculated based on the percentage of participants who either completed at least half of the program in the experimental condition, or dropped out during the experimental phase. Adherence levels ranged from 27.9% of participants (73) to 98% of participants (69) with a mean adherence percentage of 63.81%. However, caution should be exercised in the depiction of these numbers due to the lack of consensus in measuring adherence.

### Outcome Measures

Outcome measures consisted/included factors such as: depression, anxiety, social anxiety, distress, eating pattern disturbances, excessive drinking, suicidal ideation, mindfulness, self-efficacy, self-esteem, cognitive functioning, psychological inflexibility, social dysfunctioning, quality of life, rumination, emotional regulation and various other factors. Noticeable in the selected articles is that many different questionnaires (with varying validity) were used to measure a single psychological construct such as depression. For a complete overview, see Table 3.

### Key Findings

For a full overview of the overall findings, including the outcome variables, the study design, the number of participants in each study and whether human support was used in the intervention, see Table 3.

The quality of the selected articles differed considerably. Firstly, the number of participants included in the selected studies showed a wide range (from *n* = 14 to *n* = 536). Moreover, various articles did not provide data on effect sizes of significant effects (*n* = 7). In addition, some articles deconstructed or created questionnaires without reporting their psychometric properties. Lastly, some studies did not have a control group to compare the effects of the interventions to (*n* = 6). Therefore, the results are to be interpreted with caution, and the authors refrain from making conclusive comparisons between studies.

The results of the selected articles show that online preventive interventions are generally effective in reducing negative outcome measures such as depressive symptoms (*n* = 16), anxiety (*n* = 5) and stress (*n* = 6). As for the positive outcome measures, the majority of articles measuring positive health indicators showed that online preventive interventions significantly improves positive mental health factors such as well-being (*n* = 4) and social functioning (*n* = 2). However, a large part of the selected articles measuring positive health indicators also showed non-significant improvement intervention on factors such as self-efficacy (*n* = 4) and self-esteem (*n* = 4).

For studies using CBT as the intervention, small to large effect sizes were found (Cohen's *d* (*d*) between 0.36 and 1.25). Studies that combined CBT with other approaches reported small to large effect sizes (*d* = −0.17 to 0.99). CT was found to have a small effect size (*d* = 0.45). ACT interventions reported a medium effect size (*d* = 0.62 to 0.78). Mindfulness and strength-based interventions found medium to large effect sizes (*d* = 0.66 to 1.83). Cognitive bias modification found a large effect size (*d* = 0.86) and attentional bias modification a medium effect size (*d* = 0.57). The motion picture producing app found a large effect size (*d* = 0.94). For an app used as adjunct to face-to-face therapy a small to medium effect size was reported (*d* = 0.25 to 0.50). For music therapy and the decision aids the Cohen's *d* was not reported. Online interventions without human support resulted in small to large effect sizes (*d* = −0.09 to 0.99). Online interventions with robot support yielded small effect sizes (*d* = 0.37 to.44). Studies that included clinical moderation found small to large effect sizes (*d* = 0.33 to 1.25). Finally, online interventions with the combination of clinical and peer moderation found small to large effect sizes (*d* = 0.25 to 1.83).Overall, studies varied in their size, rigor of study, effectiveness and outcome measures; the effect sizes were highest for the mindfulness and strength-based intervention (1 study, *n* = 14; social functioning *d* = 1.83), CBT (*n* = 12 studies; *d* = 0.36 to 1.25), and the motion picture app (1 study, *n* = 22, no control; *d* = 0.94 depressive symptoms). Online interventions with a combination of clinical and peer moderation (*n* = 3 studies; *d* = 0.25 to 1.83) appear to result in the most stable and highest effect sizes. See Table 4 for an overview.

**Table 4:** Number of studies and effect sizes per intervention type and form of support.

**Intervention type**	***N***	**Support**	**Effect size (*d*)**
CBT	6 1 1 4	Clinical Clinical & peer Robot None	0.36–1.25 0.51–1.37 0.37–0.44 −0.76–1.03
CBT combined	1 1 3	Clinical Clinical & peer None	0.33–0.76 −0.38–0.50 −0.17–0.99
ACT	1 2	Clinical None	No effect sizes reported 0.053–0.78
Cognitive bias modification	1 2	Clinical None	No effect sizes reported −0.67–0.86
Decision aid for treatment	2 1	Clinical None	No effect sizes reported No effect sizes reported
Music therapy	1	None	No effect sizes reported
Cognitive training	1	None	0.45
Motion picture-reproducing app	1	None	0.94
Adjunct to therapy; e.g., information, registrations	1	Clinical	Ongoing
Mindfulness and strength-based intervention	1	Peer & clinical	0.66–1.83
Self-determined	1	Clinical	No effect sizes reported

*N, number of participants; ACT, acceptance and commitment therapy; CBT, cognitive behavioral therapy; CBT combined, cognitive behavioral therapy combined with other approach; d, Cohen's d*.

Even though the scope of this review was indicated prevention, 60% of the articles did not exclude participants who met criteria for a mental disorder. However, clinical stages 1a and 1b are not synonym with the absence of a mental disorder as assessed with the DSM/ICD (ref). But to give a complete overview, we summarize the 12 studies that excluded participants with a mental disorder below and in Table 5. These studies show varying results. Overall, no effects to large effect sizes were found (*d* = 0 to 1.83). Studies using CBT as the intervention found small to large effect sizes (*d* = 0.36 to >1.0). One study using an ACT intervention found no significant treatment effects, and one found significant effects (*p* = 0.027, no effect size reported). The motion picture app found a large effect size (*d* = 0.94). Mindfulness and strength-based interventions found medium to large effect sizes (*d* = 0.66 to 1.83). Online interventions without human support resulted in no effect to large effect sizes (*d* = 0 to 0.94). Studies that included clinical moderation found small to large effect sizes (*d* = 0.36 to 89). Finally, online interventions with the combination of clinical and peer moderation found medium to large effect sizes (*d* = 0.5 to 1.83). Overall, studies using mindfulness and strength-based interventions, a motion picture app or CBT found the highest effect sizes. The studies using ACT as the intervention type show varying results and no effect sizes are known for one study, making it difficult to draw conclusions on the effectiveness of ACT. Moreover, online interventions with the combination of clinical and peer moderation appear to result in the most stable (smaller range of effectiveness findings) and highest effect sizes.

**Table 5:** Study characteristics for studies excluding participants with clinical levels of symptoms.

**References**	**Screening instrument**	**Intervention type**	**Human support**	**Findings**
Alvarez-Jimenez et al. (51)	CAARMS	Mindfulness and strength based intervention	Peer and clinically moderated	Large improvement in social functioning (*d* = 1.83), subjective well-being (*d* = 0.75), strengths usage (*d* = 0.70) and mindfulness skills (*d* = 0.66) at follow-up.
van Aubel et al. (54)	Currently under treatment or need for more care assessed by psychiatrist	ACT	Clinically moderated (weekly group sessions with trained therapist)	Decrease in depressive symptoms (*p* = 0.027) compared to control. Increased mean negative affect (*p* = 0.011), relative to active controls.
Cook et al., 2019	Instrument not specified	CBT	Clinically moderated (feedback by clinicians)	Reduced risk of depression by 34% using guided i-RFCBT relative to usual care (hazard ratio = 0.66). Significant improvements in rumination, worry, and depressive symptoms.
Dickter et al. (58)	PHQ-A interview	CBT and IPT	None	Decrease in suicidal ideation (*d* = 0.60).
Mccall et al. (69)	Self-report no former diagnoses	CBT	None	Reduction in social anxiety in treatment condition (SIAS: *d* = 0.72; FNE: *d* = 0.82) then control condition (SIAS: *d* = 0.56; FNE: *d* = 0.97)
Lattie et al. (66)	Mental health history assessed by psychiatrist	CBT	Peer and clinically moderated (for guidance and technical support)	Decreased depressive symptoms ($\eta_{p^{2}}$ = 0.061) and perceived stress ($\eta_{p^{2}}$ = 0.159); significant increase of positive effect from baseline to midpoint in both groups ($\eta_{p^{2}}$ =0.321)
Levin et al. (68)	Self-report no former diagnoses	ACT	None	No differences between conditions at post or follow-up.
Simmons et al. (75)	Instrument not specified	Decision aid for treatment and life style advice, and psychoeducation	Clinically moderated (clinician present during session)	Clients were more likely to make a guideline congruent decision for treatment (93 vs. 70%; *P* = 0.004), had reduced decisional conflict and reduced depressive symptoms (follow-up: 7 points lower on PHQ).
Staples et al. (76)	PHQ-9	CBT	Clinically moderated (one condition with support clinician)	Symptom reductions on all measures at post-treatment and 3-month follow-up both conditions. Within-group effect sizes were large (*d* >1.0) and high levels of treatment satisfaction.
Takahashi et al. (77)	Self-report no former diagnoses	Motion picture-reproducing app	None	Decrease depressive symptoms (*d* = 0.94).
Topper et al. 2017	Self-report no former diagnoses + PHQ-9	CBT	Clinically moderated (weekly group session and feedback on online exercises by a therapist)	Reduced RNT (*d* = 0.53 to 0.89; 12-month follow-up: effects maintained), and symptoms of anxiety and depression (*d* = 0.36 to 0.72; 12-month follow-up: effects maintained) in both interventions. Significantly lower 12-month prevalence rate of depression and generalized anxiety disorder in both intervention groups compared to the waitlist
Traber-Walker, et al. (79)	Self-report no former diagnoses	Adjunct to therapy; for example, information, registrations	Clinically moderated (weekly individual sessions)	Ongoing

*SD, Standard Deviation; PHQ-9, Patients Health Questionnaire-9; PHQ-A, Patients Health Questionnaire-9 modified for adolescents; CAARMS, Comprehensive Assessment of At Risk Mental States; CBT, Cognitive Behavioral Therapy; IPT, Interpersonal Therapy; ACT, Acceptance and Commitment Therapy; f2f, face-to-face*.

The most robust data for the effectiveness of online preventive interventions are from the following three articles due to their high number of participants (*n-*range of studies from 413 to 536) and their RCT design. These studies provide general support for the effectiveness of online preventive interventions for youth. (1) Radomski et al. (73) used CBT as the intervention type, and did not find significant differences between conditions but user experience was significantly more positive in the intervention group compared to the control group. (2) Sanci et al. (74) researched a decision aid, and found a significantly stronger reduction in negative affect in the intervention group compared to the control group post-intervention and at the 1-month follow-up. In addition, a significant increase in help-seeking behavior was measured in the intervention group compared to the control group at the 3-month follow-up. (3) Staples et al. (76), also used CBT as the intervention type, and found significant reduction in symptoms of depression, anxiety and non-specific psychological distress with large within-group effect size (*d* >1.0), high levels of treatment satisfaction and no significant differences between the online intervention group and the routine care group.

Overall, young people commonly reported high satisfaction and usability of online interventions. For example, high levels of treatment satisfaction were reported (76). Moreover, safety, reliability (53) and positive user experience (73) of online platforms were found.

## Discussion

The focus of this review was to present an overview of indicated online preventive interventions for emerging mental health symptoms in youth (12–25 years). We aimed to identify the nature and extent of the relevant research evidence from treatment studies. This led to the following guiding question: *What is known about the use of indicated online preventive interventions for youth with emerging mental health problems?*

The findings of the included articles of the scoping review indicate the overall importance of online indicated preventive intervention. The results show that online preventive interventions are generally effective to reduce subclinical symptoms of various mental illnesses and improve several outcome measures such as quality of life and mindfulness. In addition, young people commonly reported good satisfaction, acceptability and usability of online interventions.

However, the included studies pose several limitations and therefore conclusions should be made with caution. Also the research published to date has focused predominantly on specific diagnostic categories, suggesting there is a lack of studies that have targeted transdiagnostic mechanisms. Finally, clear definitions of- as well as instruments to measure- emerging or subclinical mental health symptoms are missing. In the next section, the found gaps in the research, and the limitations of this scoping review will be discussed, as well as recommendations for future research.

### Gaps in the Literature

Overall, the included articles show that online indicated preventive mental health interventions for youth with emerging mental health issues show promise in reducing various mental health complaints, and increasing positive mental health indicators such as well-being and resilience. From the 30 articles selected for our scoping, the vast majority of the included studies were RCTs with adequate use of control groups (*n* = 24). Nonetheless, the included studies showed important shortcomings. For example, effect sizes were often not reported, psychometric qualities of used instruments were not investigated, and control groups were missing. Moreover, it remains unclear how long these positive effects last. The majority of articles had no follow-up data exceeding 3 months, and only four articles had follow-up data exceeding 7 months. The limited availability of long-term data is an issue, since it does not provide adequate insight whether online indicated preventive interventions for youth with emerging mental health issues delays the onset of a consequent mental illness, or whether it prevents the onset altogether. To provide an answer to this issue, future research regarding online indicated preventive interventions needs to investigate the long-term effects.

We note that there is an emerging consensus among researchers of the potential importance of indicated preventive interventions for young people. However, clear definitions of subclinical mental health complaints vs. clinical mental health disorders are missing, as well as instruments to measure these different stages. For example, in a considerable number of studies participants were only asked whether they were ever formally diagnosed with a mental illness. Thus, the external validity of available indicated prevention research is limited, and the findings of the studies should be interpreted with caution. The clinical staging model of McGorry et al. (40) might offer a way to differentiate subclinical mental health complaints from clinical mental health disorders using different stages of mental health disorders. The model provides clear descriptions and cut-offs [e.g., (81)]. To the best of our knowledge a clinical instrument to operationalize these stages has not yet been developed. When looked at online indicated prevention research, it becomes clear that the clinical staging model has not been fully implemented and that there is critical fundamental work still to be undertaken. Additionally, indicated prevention in terms of the clinical staging model entails the prevention of severe mental health conditions. Light mental disorders in the affective spectrum would fall in stage 1b (40). In other words, the clinical staging model does not have the same cut offs as the DSM/ICD categories. This also shows that it is relatively difficult to apply the clinical staging model at the current moment. An interesting finding from studies in at-risk populations is that emerging mental health complaints are often diffuse and non-diagnosis specific. Also, emerging complaints have divergent trajectories, potentially leading to different mental disorders as well as remission or recovery (40). Within the included research of this scoping review, however, the focus lies almost exclusively on specific disorder categories, for example, youth with depressive symptoms. Moreover, the interventions used were disorder-oriented and less individually tailored. Since emerging complaints are often diffuse, have divergent trajectories, and are underpinned by overlapping mechanisms, a transdiagnostic approach would potentially make indicated preventive interventions more useful.

Only a small proportion of the found articles focused on youth within the age of 12–25. Most studies either looked at children younger than 18 years old, or at adults above 18 years old. This finding highlights a common obstacle in the modern day psychiatry, namely the gap that exists between child and adult psychiatry. The transition from child to adult psychiatry holds a risk for disruption in continuity of care (82, 83). Despite this, the onset of disorders (5, 6), as well as the strongest health burden (6) and multilevel life transitions (82) lie within this period. The group of youth between the ages of 12–25 years old is traditionally being divided in two groups based on age, labeled ‘child’ and “adult,” while the characteristics and complaints of these individuals might suggest treating this group as a whole. More and more this need is emphasized, and currently being implemented in for example the Dutch health care system (84). The mean age of the sample of this scoping review was 18 years old, right at this cut, which also emphasizes the need to lift this boundary in scientific research and clinical practice.

To date a range of different platforms, websites and apps for online selective preventive interventions have been developed. Most studies used evidence-based therapies or frameworks for these programs. There was a great variance in the inclusion of additional human support to these interventions; ranging from sending reminders to weekly therapy sessions with a clinician. One study found no beneficial effect of inclusion of human support (76); however there is extensive research that adding human support enhances clinical effectiveness of online interventions (85, 86). The additional value of different types of human support should be investigated more extensively to be able to draw firm conclusions.

The mean adherence to the included programs varied substantially among the included studies. However, caution in interpreting this data is advised for various reasons. The biggest issue with interpreting the adherence rates in the present scoping review stems from the fact that the included papers were not using a standard method to describe the adherence to their program: therefore, there is no clear convention or determining “adherence.” For example, adherence could be described by using the program in the experimental condition “at least once (59),” or “completing at least one module (out of a total of 4 modules (57).” Consequently, rates may seem artificially high due to the unclear demarcation of “adherence.” Beintner et al. (87) found that out of a total of 216 publications that measured adherence in their analysis, 23 (10.6%) used one metric, 46 (21.3%) used two, 56 (25.9%) used three, and 63 (29.2%) included the use of four or more metrics. Indeed, it is a challenge to compare adherence rates in different studies with each other due to a missing common standard, as concluded by Beintner et al. (87). A possible solution to this methodological challenge is to introduce a-priori measurements to generate more meaningful data in future studies measuring adherence to and use of online programs, in accordance with the reasoning of Alvarez-Jiménez et al. (88). Additionally, adherence rates may be artificially inflated due to recruitment setting or participation incentive as described in the study of Mccall et al. (69) (98% adherence rate) where student participants received extra course credit contingent on the amount of modules they completed and must be taken into account when interpreting data.

The majority of studies measuring usability and acceptability (defined by how intuitive and easy a program was to use and whether the program was satisfactory and acceptable, respectively) reported acceptable levels [e.g., (57, 60, 61, 66, 68)]. However, as it was critically noted by Deady et al. (57), the largely unguided nature of online preventative interventions might negatively impact adherence. About half of the studies included in this scoping review made use of some form of human support. However, it is difficult to make inferences about the possible impact of human support on adherence since the inclusion of human support as well as the measurements of adherence varied substantially among the included studies. It is important to carefully examine the advantages of providing largely unguided interventions with low adherence vs. interventions that require more guidance but yield higher adherence rates. Moreover, it could be valuable to investigate new ways to increase adherence in unguided settings.

## Limitations and Strengths

Several limitations and strengths to this review should be highlighted. First of all, this study is a scoping review as opposed to a systematic review. Although this review has an ambitious breadth, it is not meant to be exhaustive in nature. For example, only three databases were searched. However, the advantage of a scoping review is that it aims to map key concepts, main sources, types of evidence available, volume, nature and characteristics rapidly, especially in areas where less research has been conducted. In contrast to most systematic reviews, the scoping review included not only RCTs, but different methods and study designs, implicating that the literature is potentially described more broadly (50). In addition, a scoping review is descriptive in nature (50), and no quality assessment of studies has been done. Therefore, further mechanisms and quality of evidence could not be provided.

Another limitation is that studies with certain disorder categories were excluded, for example eating disorders. One could argue this disorder would justify inclusion. Moreover, even though clear inclusion and exclusion criteria were drafted a priori, it was difficult to apply these criteria with a high degree of precision. Existing studies were often not explicitly based on concepts of clinical staging. Furthermore, the majority of the studies treated youth between the ages of 12 and 25 as two separate groups, contrary to our conceptualization of treating this age group as a whole. As a result, the identified articles varied in nature, population, methods, definitions and outcome measures, making it difficult to draw conclusions about online indicated preventive interventions for youth.

A strength of this review is that we used a transparent methodological framework to find key trends in the literature, which potentially gives a preliminary basis for future systematic reviews. Further, the review identified important gaps in the existing literature. Lastly, to our knowledge, this review is the first in the past 5 years to shed light on indicated preventive mental health intervention for youth.

## Future Research

Overall, high-quality investigations of the effectiveness of online indicated preventive interventions with follow-up data exceeding a few months for youth are missing. Further good quality research is needed to assess the effectiveness of the different online interventions using different therapeutic approaches. We suggest that researchers develop standardized definitions and instruments concerning subclinical symptoms in addition to clear definitions of “adherence.” Moreover, a gap in transdiagnostic approaches is evident; as well as research that has specifically targeted the adolescent population in the age range from 12 to 25 years, which crosses the divide between the child and adult mental healthcare systems. Future research should include clinical trials of indicated preventive interventions for youth between the ages of 12 and 25 based on the clinical staging model with a focus on transdiagnostic mechanisms.

## Recommendations

In order for effective online interventions to be implemented in large numbers of youth with emerging mental health issues, it is the authors' opinion that representatives of this youth should be involved in the development process of the interventions and the online platform (e.g., co-creation). The platform should be adaptive and improve continuously in response to feedback, thereby enabling idiosyncratic or personalized support. The review also shows that there are many different platforms and online interventions. Uniformity could prevent reinventing the wheel and contribute to the improvement of quality over time, both of the interventions and platforms, through research and the sharing of experiences. Lastly, it is of importance to that these services are financially compensated on a structural basis, for example from governments to enable ongoing innovation and development and keep up with the fast pace of development of technology. This requires commitment of governments and participation of “offline” (in-person) care parties to improve blended online and offline care adjusted to the needs of young people at different time points during their development.

## Author Contributions

MD, DN, ME, and KA completed initial study design. MD, ME, LN, KA, and ER assessed the provided articles. MD, ME, and LN made the final inclusion selection. DN, JDa, CM, AP, and TvA provided an expertise assessment and contributed extra articles to the scoping. The manuscript was written by MD, ME, and LN. All authors read and approved the final manuscript.

## Conflict of Interest

The authors declare that the research was conducted in the absence of any commercial or financial relationships that could be construed as a potential conflict of interest.
