# Supplementary material for: Online Indicated Preventive Mental Health Interventions for Youth: A Scoping Review
Source: Front Psychiatry. 2021 Apr 29;12:580843. doi: 10.3389/fpsyt.2021.580843 (PMC8116558; doi:10.3389/fpsyt.2021.580843)
Supplement: Supplementary file 1 [file Data_Sheet_1.docx]

Appendix 1.
*Search appendix*

|  | **Ovid MEDLINE(R) ALL <1946 to March 30, 2020>**  **Search date: 31 March 2020** |  |
| --- | --- | --- |
| **#** | **Searches** | **Results** |
| 1 | adolescent/ or minors/ or schools/ or universities/ or exp puberty/ | 2058371 |
| 2 | (youngster or pubert* or pubescent or prepubescent or highschool* or kid or kids or underage* or youth? or boy or boys or girl? or sibbling* or child or children or adolescent? or adolescence or juvenile or minors or teen or teens or teenager* or p?ediatric? or student? or young adult*).ab,kf,ti. | 2045906 |
| 3 | (child or p?editric? or adolescents or adolescence or juvenile).jw. | 102552 |
| 4 | (("12" or "13" or "14" or "15" or "16" or "17" or "18" or "19" or "20" or "21" or "22" or "23" or "24" or "25") adj1 (age? or yr? or year?)).ab. | 791682 |
| 5 | or/1-4 [12 - 25 yrs] | 3801919 |
| 6 | "clinical trials as topic"/ or random allocation/ | 278516 |
| 7 | (intervention or therap* or treat* or counseling or referral).ab,kf,ti. | 7189721 |
| 8 | (clinical trial or randomized controlled trial).ab,kf,pt,ti. | 914622 |
| 9 | or/6-8 [interventions] | 7640378 |
| 10 | secondary prevention/ | 20010 |
| 11 | (prevention or early therap* or early intervention?).ab,kf,ti. | 576328 |
| 12 | ("at risk" and (therap* or intervention? or treat*)).ab,kf,ti. | 78394 |
| 13 | ((emotion* or psychiatric* or psychologic*) adj3 (difficulties or distress or problem?)).ab,kf,ti. | 50299 |
| 14 | (depress* adj3 symptom?).ab,kf,ti. | 69543 |
| 15 | ((mild or moderate or onset or symptom?) adj2 (distress or stress or anxiety or depression)).ab,kf,ti. | 54193 |
| 16 | (problem? and distress).ab,kf,ti. | 12474 |
| 17 | or/10-16 [secondary prevention] | 791576 |
| 18 | telemedicine/ or exp internet/ or electronic mail/ or exp computers/ | 172371 |
| 19 | (online or ehealth or e-health or electronic health or internet or web based or web page? or blog or blogger? or blogging or weblog* or mobile health or mhealth or m health or telemedicine or virtual community or social media or chat or forum or computer* or email* or e-mail* or electronic mail or online communit* or online social network?).ab,kf,ti. | 528105 |
| 20 | 18 or 19 [online - web based - computerized] | 609607 |
| 21 | and/5,9,17,20 | 5851 |
| 22 | limit 21 to yr="2013-current" | 4054 |
| 23 | ("Internet-delivered Cognitive Behavioural Therapy for Adolescents With Anxiety" or "Evaluating an Internet-based Program for Anxious Adolescents" or "Primary Care Internet-Based Depression Prevention for Adolescents" or "CATCH-IT" or "Internet-based Program for Prevention and Early Intervention of Adolescent Depression" or "An Internet-based Program for Prevention and Early Intervention of Adolescent Depression" or "Adapted and Translated, Adolescent Depression, Internet Intervention" or "Cognitive Remediation in Youth at Risk of Serious Mental Illness" or "The Reducing Risk Study" or "Comprehensive Program for Youth Mental Health" or (EMPATHY adj1 (study or trial)) or "Rumination-focused CBT Training for the Prevention of Depression and Anxiety" or "Evaluation of an app to support emotional wellbeing of adolescents experiencing depression and/or anxiety" or "Prevention of depression and anxiety in adolescents through the Internet").ab,kf,ti. | 105 |
| 24 | (NCT03441490 or NCT02970734 or NCT01228890 or NCT03047512 or NCT02780232 or NCT01783652 or NCT02582528 or NCT03665337 or NCT02169960 or NCT01223677 or NCT03521388 or ACTRN12618001877279 or NTR1322).ab,kf,ti. | 4 |
| 25 | 23 or 24 [relevant trials] | 109 |
| 26 | 22 or 25 | 4148 |
| 27 | remove duplicates from 26 | **4101** |
|  | **PsycINFO <1806 to March 30, 2020>**  **Search date: 31 March 2020** |  |
| **#** | **Searches** | **Results** |
| 1 | exp college students/ or schools/ or colleges/ or puberty/ | 127583 |
| 2 | (youngster or pubert* or pubescent or prepubescent or highschool* or kid or kids or underage* or youth? or boy or boys or girl? or sibbling* or child or children or adolescent? or adolescence or juvenile or minors or teen or teens or teenager* or p?ediatric? or student? or young adult*).ab,id,ti. | 1359828 |
| 3 | (child or p?editric? or adolescents or adolescence or juvenile).jx. | 115613 |
| 4 | (("12" or "13" or "14" or "15" or "16" or "17" or "18" or "19" or "20" or "21" or "22" or "23" or "24" or "25") adj1 (age? or yr? or year?)).ab. | 297519 |
| 5 | ("200" or "320").ag. | 814364 |
| 6 | or/1-5 [12 - 25 yrs] | 1854686 |
| 7 | intervention/ or early intervention/ or group intervention/ or school based intervention/ or clinical trials/ | 103162 |
| 8 | (intervention or therap* or treat* or counseling or referral).ab,id,ti. | 1130067 |
| 9 | (clinical trial or randomized controlled trial).ab,id,ti. | 31480 |
| 10 | or/7-9 [interventions] | 1149329 |
| 11 | prevention/ or primary mental health prevention/ or relapse prevention/ or suicide prevention/ | 39164 |
| 12 | (prevention or early therap* or early intervention?).ab,id,ti. | 136605 |
| 13 | ("at risk" and (therap* or intervention? or treat*)).ab,id,ti. | 25635 |
| 14 | ((emotion* or psychiatric* or psychologic*) adj3 (difficulties or distress or problem?)).ab,id,ti. | 59716 |
| 15 | (depress* adj3 symptom?).ab,id,ti. | 63974 |
| 16 | ((mild or moderate or onset or symptom?) adj2 (distress or stress or anxiety or depression)).ab,id,ti. | 46307 |
| 17 | (problem? and distress).ab,id,ti. | 11638 |
| 18 | or/11-17 [secondary prevention] | 296559 |
| 19 | telemedicine/ or exp internet/ or online therapy/ or exp websites/ or online community/ or online social networks/ | 44831 |
| 20 | (online or ehealth or e-health or electronic health or internet or web based or web page? or blog or blogger? or blogging or weblog* or mobile health or mhealth or m health or telemedicine or virtual community or social media or chat or forum or computer* or email* or e-mail* or electronic mail or online communit* or online social network?).ab,id,ti. | 222274 |
| 21 | 19 or 20 [online - web based - computerized] | 229194 |
| 22 | and/6,10,18,21 | 4790 |
| 23 | limit 22 to yr="2013-current" | 3348 |
| 24 | ("Internet-delivered Cognitive Behavioural Therapy for Adolescents With Anxiety" or "Evaluating an Internet-based Program for Anxious Adolescents" or "Primary Care Internet-Based Depression Prevention for Adolescents" or "CATCH-IT" or "Internet-based Program for Prevention and Early Intervention of Adolescent Depression" or "An Internet-based Program for Prevention and Early Intervention of Adolescent Depression" or "Adapted and Translated, Adolescent Depression, Internet Intervention" or "Cognitive Remediation in Youth at Risk of Serious Mental Illness" or "The Reducing Risk Study" or "Comprehensive Program for Youth Mental Health" or (EMPATHY adj1 (study or trial)) or "Rumination-focused CBT Training for the Prevention of Depression and Anxiety" or "Evaluation of an app to support emotional wellbeing of adolescents experiencing depression and/or anxiety" or "Prevention of depression and anxiety in adolescents through the Internet").ab,cn,id,ti. | 106 |
| 25 | (NCT03441490 or NCT02970734 or NCT01228890 or NCT03047512 or NCT02780232 or NCT01783652 or NCT02582528 or NCT03665337 or NCT02169960 or NCT01223677 or NCT03521388 or ACTRN12618001877279 or NTR1322).ab,id,ti. | 0 |
| 26 | 24 or 25 [relevant trials] | 106 |
| 27 | 23 or 26 | **3444** |
|  | **Scopus**  **Search date: 31 March 2020** |  |
| **#** | **Searches** | **Results** |
| 1 | TITLE-ABS-KEY ( youngster  OR  pubert*  OR  pubescent  OR  prepubescent  OR  highschool*  OR  kid  OR  kids  OR  underage*  OR  youth?  OR  boy  OR  boys  OR  girl?  OR  sibbling*  OR  child  OR  children  OR  adolescent?  OR  adolescence  OR  juvenile  OR  minors  OR  teen  OR  teens  OR  teenager*  OR  p?ediatric?  OR  student?  OR  "young adult*" )  OR  TITLE-ABS-KEY ( ( "12"  OR  "13"  OR  "14"  OR  "15"  OR  "16"  OR  "17"  OR  "18"  OR  "19"  OR  "20"  OR  "21"  OR  "22"  OR  "23"  OR  "24"  OR  "25" )  W/1  ( age?  OR  yr?  OR  year? ) )  AND  ( ( TITLE-ABS-KEY ( intervention  OR  therap*  OR  treat* ) )  OR  ( TITLE-ABS-KEY ( "clinical trial"  OR  "randomized controlled trial" ) ) )  AND  ( TITLE-ABS-KEY ( online  OR  ehealth  OR  "e-health"  OR  "electronic health"  OR  internet  OR  "web based"  OR  "web page?"  OR  blog  OR  blogger?  OR  blogging  OR  weblog*  OR  "mobile health"  OR  mhealth  OR  "m health"  OR  telemedicine  OR  "virtual community"  OR  "social media"  OR  chat  OR  forum  OR  computer*  OR  email*  OR  "e-mail*"  OR  "electronic mail"  OR  "online communit*"  OR  "online social network?" ) )  AND  ( ( TITLE-ABS-KEY ( prevention  OR  "early therap*"  OR  "early intervention?" ) )  OR  ( TITLE-ABS-KEY ( "at risk"  AND  ( therap*  OR  intervention?  OR  treat* ) ) )  OR  ( TITLE-ABS-KEY ( ( emotion*  OR  psychiatric*  OR  psychologic* )  W/3  ( difficulties  OR  distress  OR  problem? ) ) )  OR  ( TITLE-ABS-KEY ( ( depress* )  W/3  symptom? ) )  OR  ( TITLE-ABS-KEY ( ( mild  OR  moderate  OR  onset  OR  symptom? )  W/2  ( distress  OR  stress  OR  anxiety  OR  depression ) ) ) )    AND  ( LIMIT-TO ( SUBJAREA ,  "PSYC" )  OR  LIMIT-TO ( SUBJAREA ,  "SOCI" )  OR  LIMIT-TO ( SUBJAREA ,  "NURS" )  OR  LIMIT-TO ( SUBJAREA ,  "COMP" ) ) | **3577** |
